# Challenges of Access to WASH in Schools in Low- and Middle-Income Countries: Case Study from Rural Central Kazakhstan

**DOI:** 10.3390/ijerph18189652

**Published:** 2021-09-13

**Authors:** Zhanerke Bolatova, Kamshat Tussupova, Berik Toleubekov, Kanat Sukhanberdiyev, Kulyash Sharapatova, Martin Stafström

**Affiliations:** 1 School of Public Health, Biomedicine and Pharmacy, Karaganda Medical University, Karaganda 100008, Kazakhstan; bolatovazhanerke93@gmail.com (Z.B.); berik.toleubekov@med.lu.se (B.T.); 2 Division of Water Resources Engineering, Lund University, SE-22100 Lund, Sweden; 3 Kazakh National Agrarian University, Almaty 050010, Kazakhstan; 4 Department of Clinical Sciences, Lund University, SE-22100 Lund, Sweden; martin.stafstrom@med.lu.se; 5 Unicef, Nur-Sultan 010000, Kazakhstan; kana6@msn.com; 6 Department of Surgery and Gynecology and Pediatry, Pavlodar Branch, Semey State Medical University, Pavlodar 140000, Kazakhstan; sharapatova_kulyash@mail.ru

**Keywords:** access to water in school, access to sanitation in school, access to hygiene in school, availability of WASH, accessibility of WASH, operation of WASH, maintenance of WASH, sustainability

## Abstract

Safe water and sanitation, which give rise to appropriate hygiene, are fundamental determinants of individual and social health and well-being. Thereby, assessing and widening access to sustainable, durable water and sanitation infrastructure remains a global health issue. Rural areas are already at a disadvantage. Poor access to water, sanitation, and hygiene (WASH) can have a major negative effect on students in rural schools. Thus, the paper aims to assess the current condition and the challenge to access WASH in rural Kazakh schools. The study was conducted in three rural schools in Central Kazakhstan. Data were gathered through a survey among pupils, observations of the WASH infrastructure and maintenance, and a face-to-face interview with school administrators. The mean survey response rate was 65% across schools. Results indicated there was no alternative drinking-water source in schools, and 15% of students said they had access to water only occasionally. Half of the students reported that the water was unsafe to drink because of a poor odor, taste, or color. The toilet in school 3 was locked with a key, and a quarter of the students reported there was no access to a key. Moreover, not having gender-separated toilet facilities was a challenge because of the traditional gender norms. Despite the effective regulations and measures of handwashing taken during COVID-19, 27.7% of the students answered that soap was not offered daily in classrooms. Additionally, warm water was only provided in school 2. About 75% of students did not have access to drying materials continuously. The study shows that having the schools’ infrastructure is not enough when characteristics, such as availability, accessibility, maintenance, operation, quality of services, education, and practices, are ignored. Cooperation between local education authorities, school administration, and parents should be encouraged to the achievement of the sustainable development goals (SDGs) by 2030.

## 1. Introduction

### 1.1. Background

Safe water and sanitation, which empower appropriate hygiene, are essential determinants of well-being, quality of life, and human nobility [1]. Thereby, the lack of reliable access to a secure and sustainable WASH infrastructure combined with the behavior of hygiene remains a significant public health problem [2]. In return, the improved WASH can reduce challenges, such as the outbreak of waterborne disease and other health complications [3]. Moreover, the COVID-19 pandemic focuses on an easy, primary prevention activity, handwashing, that most people will carry out independently [4].

Besides, in 2015, the global community set a measurable target in the form of Sustainable Development Goal (SDG) 3, which aimed to ensure healthier lives and encouraging well-being at all ages to achieve sustainable growth. Additionally, SDG 4 seeks to improve the proportion of education facilities with access to an appropriate learning environment, including essential drinking water, sanitation, and hygiene [5]. Moreover, SDG 6 points out the value of cohesive WASH systems as a whole [6]. It seeks to ensure complete coverage and sustainable water and sanitation management by 2030 [7].

Additionally, World Health Organisation (WHO) and United Nations Economic Commission for Europe (UNECE) coordinated the program Joint Monitoring Program (JMP), which collects the WASH data in various facilities annually [8]. This type of monitoring enables mapping the whole WASH situation [9]. Nevertheless, in this database (JMP), there are no data about the WASH situation in Kazakh schools. Inadequacies in water and sanitation in the school environment impact children’s health and school attendance [10]. After all, pupils spend a long time at school; hence, schools have a valuable and vital role in shaping children’s health knowledge, attitudes, behaviors, and health outcomes. Therefore, one of a school’s primary functions is providing educational functions and providing children with life skills and capacities that promote their well-being [11]. Thus, the lack of data on WASH coverage in schools is an obstacle in protecting children’s rights. The presence, in conclusion, of a WASH service is an inalienable right of every person [12].

While building the school, drinking-water and sanitation points are present due to Kazakh regulation [13,14]. However, the monitoring of the water points is only conducted by the Ministry of Education. At the same time, the provision of access to WASH by each student is the responsibility of the school administration. Thus, the holistic view of the WASH, looking at the official statistics of the Ministry of Education, seems not possible while the local schools do not provide information to the society [15]. Additionally, it is unclear the method that is used for assessing the access to WASH. Moreover, rural areas are already at a permanent disadvantage. The lack of data at the international and national levels and the lack of a method for assessing access to WASH could lead to the health impairment of rural schoolchildren due to the lack of adequate access to WASH infrastructure. Thus, the system approach for data collection and analysis on access to WASH in schools is needed.

Additionally, there are no basic necessities in the rural areas of Kazakhstan. The WHO and (United Nations Children’s Fund) UNICEF indicate that the poorest fifth of Kazakhstan’s rural population has the same amount of piped water coverage as Sub-Saharan Africa [16]. Rural Kazakh schools, as institutions, could potentially mitigate these shortages. However, their current status in providing basic and essential WASH is currently unknown and thus needs to be determined. In line with this, the paper’s primary objective is to assess the current access to WASH in schools in the rural region of Central Kazakhstan.

### 1.2. Review of Methods of Assessing Access in Schools

A variety of tools to assess WASH access in schools and monitor progress has been developed by international organizations. Therefore, it is essential to choose a suitable method and survey tool, taking contextual factors, e.g., location, program objective, school type, local practices, and culture, into consideration.

Firstly, the tool “Core questions and indicators for monitoring WASH in Schools in the Sustainable Development Goals” was developed by the Joint Monitoring Program for Water and Sanitation. Moreover, this tool aims to increase surveys’ comparability over time, both between and within countries, and harmonize data with WASH SDG indicators in schools and applied in national surveys [17]. In addition, the tool “Accelerating Water and Sanitation for All Programme (ASWA) “is a multi-country program in low- and lower-middle-income countries to improve sanitation and access to hygiene” [18]. However, after a thorough assessment, both tools seemed to be less suitable, as the first tool was more assessing the general access with little insight information, and the second tool is more of a framework and dedicated towards less developed infrastructure context and required high adaptation.

The “Surveillance of water, sanitation, and hygiene in schools” tool produced by WHO and UNECE is used at various stages and for different reasons with different coverage. This tool was adapted to former Soviet Union country situations by WHO and considered more suitable, requiring minor adaptation. At the regional level within a nation (provincial, municipal, or district), responsible education or public health authorities may use it to comprehensively track different aspects of WASH distribution in schools and assess student attitudes, experiences, and behavior [19]. Based on Kazakh standards, described in Kazakhstani documents, such as Construction standards of the Republic of Kazakhstan “Educational institutions”, Code of rules of the Republic of Kazakhstan “Educational institutions”, and Sanitary Rules “Sanitary and epidemiological requirements for educational facilities”, the study was based on this instrument [13,14,20].

Furthermore, the method’s flexibility was considered an asset because it had been implemented based on current national and international norms, methodologies, and recommendations for tracking WASH in schools. Another strong point is a periodically posed sequence of questions between instruments and within each instrument. This repetition aims to reflect various facets of WASH service delivery as well as various viewpoints. Finally, it helps to have a detailed review of the findings and validate their validity [19].

Considering the information above, the additional aim of this survey is to test the WASH assessment tool “Surveillance of water, sanitation, and hygiene in schools” developed jointly by WHO and UNECE for data collection purposes.

## 2. Materials and Methods

### 2.1. Survey Design

The survey was performed in February 2020, before the COVID-19 pandemic, in three schools in three different villages in Central Kazakhstan. All tools were translated to the Kazakh language. Questionnaires and interviews were conducted in Russian and Kazakh languages. A total of 166 questionnaires were completed, 3 school administrators were interviewed, and 3 observations were conducted and included in the analysis. By Kazakh law, the school classes are divided into three: junior school (1–4 grades), middle school (5–9 grades), and high school (10–11 grades).

The authors of the survey got official consent to conduct the questionnaire from the heads of schools. At the end of the school day, the school member introduced the interviewer to pupils, and the interviewer explained the all-importance of this survey. Participants had a free choice to participate in the study, and participation was anonymized. In the questionnaire, the only private data given was age and sex. If some aspects of the questionnaire were challenging to understand, students could ask the interviewer. If participants felt uncomfortable, they did not have to answer the questions. All questions on access to water, sanitation, and hygiene concerning the young students were asked to everyone. Questions about limited mobility students were not studied in this survey because disabled students attend specialized schools. Moreover, the questions about menstrual hygiene were removed because they are not regulated by law.

Students filled it out for about 60–80 min. In schools 1 and 2, the interviewer collected filled-out questionnaires from middle and high school students (grades 7–11) at the end of the school day. The questionnaire to middle school students (grades 5–6) was collected on the following day. Junior students (grades 2–4) brought the questionnaires home to fill them out with their parents. In school 3, middle and high school students participated in the survey. However, junior students were excluded, as they could not attend school due to adverse weather conditions at that time.

A total of 166 students (85 female, 77 male) participated in the survey, with a mean age of 11.67 ± 3.14. Students were distributed, as 102 students (50 female, 48 male, and 4 students did not indicate their gender) from school 1, 35 (23 female, 12 male) from school 2, and 29 students (12 female, 17 male) from school 3. Detailed information about the participants is described in Table A1.

Subsequently, the observation of WASH facilities was conducted. The WASH facilities were observed at the end of the school day or after a break time. Moreover, photos and videos were taken. Photographing and videotaping in rural schools during observations of water, sanitation, and hygiene infrastructure were allowed with the consent and permission of school administration.

A face-to-face interview with the school administrator was conducted in the respondent’s office. Standardized questions regarding the type of school program, the number of school staff and pupils, their separation by gender, budget, the person responsible for operation and maintenance, and WASH characteristics were used. The respondents were in some instances given answer alternatives for some questions and were given open-ended answers for some. The interviews lasted between 60–80 min. The interviewer tried to get all the answers to the questions.

### 2.2. Description of the Area and Schools

The study was carried out in a rural district of the Central Kazakhstan region. The climate is continental, with cold winters and hot and dry summers. In January, the average temperatures range from −16 to −17 °C and in July from +20 to +21 °C. The rural district has three villages. In every village, there is an educational institution. All schools are public, and school programs are about 7–10 h. The schools have 2 shifts (morning and afternoon). Schools 2 and 3 are Kazakh, while school 1 is mixed (Russian and Kazakh). School 1 is the largest of them, with 182 pupils. The total number of staff is 66. Forty-two of them are teachers, 6 are administrative staff, and 18 are operation and maintenance staff. Additionally, only this school has its school canteen. The second-biggest school is school 3, where 58 pupils are enrolled. The total number of staff is forty-five; seventeen of them are teachers (37.8%), four of them are administrative staff and teachers (8.9%), and twenty-five people are operations and maintenance staff (55.6%). WASH services were renovated in 2018. Moreover, the schools provide classes from the first till the eleventh grade, except school 2. In that school, there are 40 pupils only, with students in grades 1–9. The total number of staff is thirty, and fourteen of them are teachers (46.7%), three administrative staff (10%), and thirteen operations and maintenance staff (43.3%). WASH services were renovated in 2019. School characteristics are described in Table A2.

### 2.3. WASH Assessment Elements

Drinking water performs various essential roles that help the body function, including temperature control, preservation of delicate tissues, transport of nutrients, and waste disposal [21].

Sanitation provides facilities and services for preventing contact with human urine and excreta [22].

Hygiene refers to habits that can enhance cleanliness and promote health, such as daily handwashing, face washing, soap, and water bathing [22].

**Figure 1:**
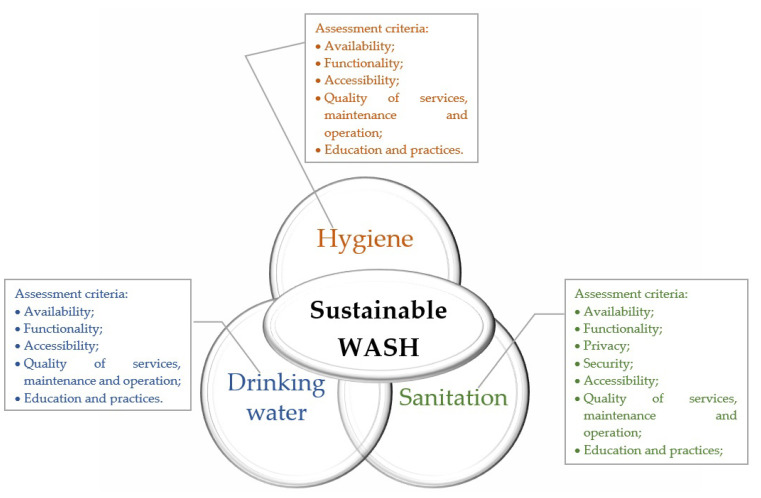
System approach to WASH.

A sustainable school WASH system should meet availability, functionality, accessibility, privacy, operation and maintenance, education, and practices (Figure 1. System approach to WASH).

Availability and functionality. The WASH facilities’ construction is not broken; service is delivered efficiently [23]. As for toilets, the toilet doors are always opened, or if they are closed by key, the key should always be available [24].

Accessibility. WASH facilities must be easily reached and located not too far away, age-friendly, and disability-friendly [23,24]. Children can use equipment individually with little effort [17].

Privacy. The toilets must close from the inside and without holes in the facility’s whole construction [24].

Quality of services, operation, and maintenance. WASH equipment works correctly, and adequate quality requirements are met [23]. In addition, assurances of cleanliness and availability of supplies, particularly during peak periods of use of WASH facilities, are met [19,25].

Education and practices. School staff and teachers may play an essential role in promoting safe habits among pupils through teaching and setting a good example [19].

Data collection with the triangulation technique is effective and beneficial. The interview with the school administration gives perspective on the WASH system and the types of services provided to students. The observation stage may identify problems that are hidden under the surface. A pupil questionnaire reveals children’s perceptions of the WASH service. The triangulation details are available in Table A3: Data analysis triangulation.

Table A1, Table A2 and Table A3 facilitates appropriate data gathering using the data triangulation approach. The table shows the questions and methods, and it is possible to see which method was used to achieve a particular question. The water availability criterion will be used as an example for the proper use of an Table A3. The table shows that data, such as the primary source of water, water availability over the school year, and additional water resources, were gathered through an interview with the school administration. The students’ questionnaire included information, such as the availability of water for drinking and handwashing purposes at school and places obtaining water for learners. The observation was done to get a complete picture, and the following questions were answered: primary water resource, availability of water, amount of drinking-water points, and location of drinking-water points. Other WASH system criteria can be viewed in Appendix A in this manner as well.

## 3. Results and Discussion

### 3.1. Drinking Water

#### 3.1.1. Water: Availability and Functionality

During the interview, it was defined that schools’ primary drinking-water source was piped water supply into the school building in schools 1 and 2. The piped water supply is potable water that is supplied directly to the customer. A pipeline network transfers it from its collection point to tap customers. Much of the time, this water is made potable by a treatment center and disinfection and then deposited in one or more storage tanks until it is used. It usually comes from groundwater, a canal, or an obvious source, and it goes through a number of treatments before meeting the user. Water is pumped or gravity fed naturally to the treatment plant from its collecting point (well, canal, or source).

However, in school 3, it was sourced from its borehole. The borehole is a tubular well that may reach depths of over 100 m. Their organization is carried out with the assistance of casing pipes. A pump with a clogging-resistant filter is used to raise the water. The interview defined that the drinking water from the main source was always available throughout the school year. The water from these sources was used for drinking, personal hygiene (including handwashing, showering), cooking, cleaning, and laundry. There were no secondary sources of water; if the main water resource was out of order, the school did not have an alternative source of drinking water. Based on reports from United Nations Children’s Fund and World Health Organization, these types of water sources are safe and improved [17]. However, the lack of an alternative supply of drinking water does not guarantee well-being. The cases of water cut-offs create obstacles for the students to keep hydrated, maintain hand hygiene, and for the maintenance staff to maintain a clean school environment, which may be dangerous to the children. The absence of this practice can lead to the spread of infection among students and staff.

Furthermore, drinking water was available in all these schools at the time of observation. During the checking, the water ran from the primary water source. Nevertheless, the water pressure was weak in school 3, where the borehole was the primary source. The number of functional drinking-water points was six, one, and three in schools 1, 2, and 3, respectively. All of them were for the use of pupils and not broken. Moreover, all points were age-friendly and available even for the smallest pupil at school. It was observed that drinking-water points were placed on each school building’s floor, inside and outside the toilet facilities (classes), and near the canteen (school 1).

A total of 15.7% of students answered that they never drank water at school time. Although, constant, flexible water gives pupils confidence that they stay hydrated and can study without thirst [26]. Drinking water is essential for students’ well-being; the absence of water during school affects the study process and health [24]. Nevertheless, 15% of pupils reported that water was rarely or never available. Furthermore, the percentage of negative answers in school 3 was higher than in schools 1 and 2, where a borehole was the source of drinking water even though well-hydrated students study better due to improved memory, attention, and concentration [27,28]. Almost every third student (28.9%) reported that they drank the school’s water. Moreover, of these, more than half of students studied at school 3. It showed that students had a greater trust in a centralized water supply than in a decentralized one. Moreover, the drinking water came from the tap, so students might perceive it was as less safe. A total of 35.5% of the students did not get water from the school. They brought the water from home or bought it from the canteen in school or outside shops. Moreover, it was essential that those students would be provided with continuous water for the effectiveness of hygiene promotion; 75.3% of students answered that water was always available and most of the time.

#### 3.1.2. Water: Physical Accessibility

It was observed that drinking water points were age-friendly. Even though drinking water had to be available to everyone, including small or weak students, only more than half of students reported that the pupils in the lower grades could obtain water by themselves. Moreover, every fifth pupil at school 3 reported it. This question was directed to everyone, including high school students. Additionally, 56.7% of students who confirmed this information were from 2–6 grades.

#### 3.1.3. Water: Quality of Services, Operation, and Maintenance

During the interview, it was revealed that the functional primary drinking water source provided enough water for the school’s needs, such as water for drinking, personal hygiene, food preparation, cleaning, and laundry. The primary water source’s quality was tested on the school premises to comply with national drinking water standards in the past 12 months. Protocol of laboratory study of water from a primary source was tested by the National Centre of Expertise of the Committee of Sanitary and Epidemiological Control of the Ministry of Health of the Republic of Kazakhstan. It contained bacteriological and physic-chemical examination. The last part included turbidity, color, odor, pH, common iron, acidification, and hardness. The bacteriological examination studied microbial number, total coliform bacteria, and thermotolerant coliform bacteria. The test result showed that all basic indicators of water proved the quality of drinking water to be acceptable. Moreover, school staff reported the absence of complaints about the quality or availability of drinking water in the school in the past 12 months. School management and staff were responsible for identifying and reporting issues with drinking-water systems and facilities to operate and maintain the drinking water provision on the school premises.

It was observed that area around the water point was clean and was free from dirt and contamination. There was no damage to the water point. All water sources were protected with corresponding means. All fundamental indicators of water had to correspond to the quality of drinking water. Therefore, all water points were without color, odor, and taste.

Nevertheless, 29.5% of students assessed the water as unpotable because of bad smell, taste, and even color. A total of 9% and 15.1% of students complained about the far distance and crowdness, respectively. Additionally, 7.8% of students reported that drinking water points were dirty or broken, and 11.4% of students were too shy to ask permission to drink water. Another 12.7% of students complained that they were not allowed to drink water during class time.

### 3.2. Sanitation

#### 3.2.1. Sanitation: Availability and Functionality

The number of available, functional, and gender-free flush toilets was six in school 1 and two in schools 2 and 3. Toilets were suitable even for young students. All toilets were in the building and freely accessible except for school 3, where both toilets had locks from outside. When a student felt the need to use the toilet during class, he or she asked the teacher for permission to go out. It was revealed that students had to ask for the key from the janitor. The janitor sat in the corridor, nearby to the entrance. This person sat in his place all the time as a watchman. All students, regardless of age, asked for the key from a janitor. Students could reach the janitor easily because the building of the school was a small and one-story building. After taking the keys from the janitor and using the toilet, students locked the door with the keys and returned them to the janitor. A quarter of the students in this school reported that they did not have access to the key. Consequently, they avoided using them and risked severe health problems and lack of concentration during the school day.

Furthermore, 76.5% of students answered that they visited the toilet at school whenever they needed to. Another 11.4% of students did it only when they absolutely could not wait anymore.

Additionally, only school 1 had a separate toilet for school staff and an additional toilet cubicle outside the building. Kazakhstani sanitary rules refer to at least one latrine for 20 girls and one latrine for 30 boys [20]. All the schools met this requirement. However, school 1 had a special condition: one of the toilets was outside, and the accessibility seemed limited to that toilet during the year. It was not possible to assess the toilet conditions, as it was outside, and it was −40 °C, and the toilet was under the snow, which proved the limited accessibility of students to the toilet during a certain period of the year. The path used to access it had not been cleared from snow. As winter in the area lasts five months, from November to April, this is the most relevant challenge in Central Kazakhstan. That is, during these months, students experience an acute shortage of toilets.

Moreover, the use of outside toilets was associated with a risk of hypothermia, as children go straight from a warm classroom to a toilet in the cold. In addition, it was a very uncomfortable situation, resulting in children trying to run home in order just to go to the toilet. Therefore, it could potentially have detrimental effects on their genitourinary system and their psychological state. It was especially dangerous for girls because hypothermia could have effects on their child-bearing abilities. Additionally, in schools 2 and 3, two toilet facilities were located in one single room. Given the local mentality, girls hesitated to go to the toilet if there were boys in the neighboring toilet room. Furthermore, some pupils were embarrassed to use the toilet with a same-sex pupil and the opposite sex. Additionally, there was a risk of developing shy bladder syndrome. Paruresis, also known as shy bladder syndrome, is a social anxiety condition that involves concern and avoidance of urinating in public settings [29]. According to the Kuoch et al. systematic review, the prevalence of paruresis ranged between 2.8 and 16.4 percent [30].

#### 3.2.2. Sanitation: Accessibility, Privacy, and Security

During the interview, it was defined that enough privacy was provided in toilet cubicles. Pupils’ toilets were functional during the academic year, and there were no issues with their functionality. All toilets were characterized as accessible to all pupils. In schools 1 and 3, pupils visited the toilet anytime; however, they had to ask permission during class. Nevertheless, in school 2, pupils were free to use the toilets during the school day.

All toilets were gender-neutral. There was no sign on the door that indicated the gender. One-third of students assessed that not having gender-specific toilets was a problem; out of them, 57.1% of them were girls, and 40.8% were boys. Moreover, the analysis showed that half of them were students of the junior classes. Young and small children felt uneasy since there was practically no privacy in the common restrooms. Some children purposefully did not drink water or ate at school so that they did not have to use the restroom. Gender-neutral toilets led to inconvenience while in use.

Moreover, 10.8% of students indicated that their classmates had frequently encountered trouble, and 10.2% of them reported their bad experiences. The questionnaire asked everyone whether or not the youngest pupil had access to the toilet. Every fifth pupil reported that the youngest pupils could not use the toilet without any help. Additionally, 64% of them were students of junior classes, and 36% were from middle and high school. Out of the total, 64% of junior school students who answered that they could not use the toilet were girls. Furthermore, girls could be too shy if someone saw who was in the toilet. As the toilets were unsuitable for younger school children, they were encouraged to endure until they returned home.

#### 3.2.3. Sanitation: Quality of Services, Operation, and Maintenance

All school staff said that toilet facilities were cleaned twice per day or whenever needed, and there was enough lighting and ventilation inside. All toilets were heated during the cold weather. Additionally, culturally appropriate means for anal cleansing were always available. In the toilet facilities, the general waste bins were provided and emptied on time.

All toilet facilities were clean, and there were functional lighting and adequate ventilation during the observation time. It was examined that in schools 1 and 2 that a cleaning schedule was posted on the toilet door. In school 3, no such schedule was on display. Yet, 11.8% of pupils assessed the toilets to be rarely clean and never clean. Furthermore, 75% of them were students who visited the toilet whenever they needed to, 14% were pupils who visited the toilet when they could not hold anymore, and 11% were students who never visited the toilet. On the other hand, 72.9% of students reported that there was always enough light in the toilet facilities. Half of the students (49.4%) assessed the toilet facilities as excellent; moreover, 30.7% assessed it as okay.

Moreover, almost half of the students (42.2%) reported that they could never find toilet paper. Of them, 89.9% were students who visited the toilet whenever they needed to and when they could not hold anymore. The absence of toilet paper was a key reason for not using the school toilet. It, in turn, may lead to health problems for pupils. Although these people used the toilet, still, it had to be a limiting factor to use the toilet free anytime they wished.

### 3.3. Hygiene

#### 3.3.1. Hygiene: Availability and Functionality, Accessibility

The observation by authors showed that the total number of handwashing facilities was six, one, and three at schools 1, 2, and 3, respectively. In these schools, the handwashing facilities were located near the toilet facilities and in classrooms and also nearby to the canteen in school 1. All of them were functional and not broken. The number of functional handwashing facilities with soap was three in school 1 and one in school 2. Nevertheless, from observation in school 3, there was no soap. Washing hands with soap is the most effective way to reduce infectious diseases, especially during the pandemic situation, such as COVID-19. Half of the students faced continuous access to handwashing facilities all the time, while every third showed a lack of access to soap. During the observation, all schools did not have hot water; however, one of them had a boiler to provide warm water for pupils. The absence of hot water at school might affect healthy behavior, like handwashing, especially in wintertime. The school must not have only soap and water; it is also crucial to be provided with drying materials. Furthermore, every fifth student (24.1%) reported that drying materials were always available or almost always. Moreover, it was observed that disposable paper towels were provided in schools 1 and 2. The third school did not have any drying facilities. An equally important aspect of hygiene promotion is drying materials, as it helps prevent infectious diseases. Nevertheless, this aspect suffered from a lack of provision.

Handwashing is a popular measure for preventing infectious diseases. However, only half of the students responded that everyone at their school washed their hands after going to the toilet.

The majority of the students (53.6%) reported that the youngest and smallest pupils could use hand-washing facilities without any help. Of them, 66% were middle and high school students. A total of 23.5% denied this statement, and 71.7% of them were students from junior school. The strategically incorrect size and availability of handwashing facilities can be an obstacle to practice healthy behavior.

#### 3.3.2. Hygiene: Quality of Services, Operation, and Maintenance

It is crucial to keep the handwashing facility clean and functional. For students’ safety, records of the maintenance and cleaning were observed in schools 1 and 2. The kept records were satisfactory because the responsible person for cleaning put the sign, the date, and time of the last cleaning. Nevertheless, only 27.1% of students assessed hand-washing facilities as excellent and okay. It showed a low level of satisfaction. Moreover, dissatisfaction with facilities could be an obstacle to promote proper hygiene behavior. The dirty handwashing facilities could be an obstacle to the pupil to wash their hands; however, all of them were clean. Additionally, it is crucial to be provided with drying materials. Nevertheless, disposable paper towels were provided only in schools 1 and 2. School 3 did not have any drying facilities.

### 3.4. WASH: Education and Practices

This chapter examines the issues of educational practice in the school concerning teaching children about the importance of drinking water, sanitation, and personal hygiene, particularly handwashing. The second aspect is what practices have developed in the school based on the opinion of students, and the third aspect is what practices students themselves use in regards to access to water, sanitation, and hygiene.

School staff claimed that pupils could drink water at school whenever they needed it, including during classes. Pupils got the drinking water from taps outside of the toilet facilities, at the canteen free of charge, and they brought water from home. However, 17.5% of students never drank water at school, with a greater proportion (about 1/5) at school 3. Moreover, no informational materials about drinking water were provided. After all, educational posters and reminders could help pupils remember to drink, as hydration could help them stay focused and not worry about thirst during the school day. The other limitation showed that 22.3% of respondents had limited access to drinking water only at a specific time, such as breaks, after class, and lunch breaks. To break this limitation, students had to ask permission to drink water from teachers. The percentage of negative answers in school 1 was higher than in other schools. Children should not have to ask for permission to drink water, and school staff should encourage students to stay hydrated. Restrictions in water access may lead to the absence of attention during classes and risks for pupils’ well-being [31]. Although the school staff could be a positive force to empower hydration throughout the school day, only half of the students (47%) reported that they talked about the importance of drinking water. The facilitation by educators is crucially important and on par with the accessibility and availability of water [27]. Almost more than half of students said that they had access to the toilet at any time in school, every third student thought that they could use the toilet during breaks before or after classes but not during classes, and 6% said that it required the teacher’s permission. Those who limited themselves in that time frame or were limited by teachers’ permission had to understand that they were at risk of having health issues. Refraining from using the toilet facilities can lead to health problems, such as urinary infections. All interviewees noted that they had not ever reported episodes of bullying or violence in the school facilities. Additionally, there was a complaint procedure for all students to report issues in school toilets. Action was always taken for complaints about toilet issues. Half of the students (48.8%) answered that they were educated about sanitation hygiene and proper hygiene behaviors when using toilets at school. However, it is necessary to conduct educational work among children about the rules of using toilets and personal hygiene products to prevent diseases. Moreover, educational posters promoting healthy and hygienic use of toilets were only on display in schools 1 and 2.

It was observed that the visual educational materials about handwashing were available in schools 1 and 2. Educational materials improve knowledge among pupils about hand hygiene. Moreover, it prevents infectious diseases by reducing contamination through dirty hands. Additionally, it empowers students to wash their hands. Despite this, only 59.6% of students washed their hands whenever they were dirty, 62.7% practiced handwashing before eating, 66.3% after using the toilet, 57.2% after playing with pets, 40.4% after contact with friends who were not feeling well, 53% after using public transport, and 66.9% after coming back home. A total of 63.9% of students practiced handwashing with water and soap and 28.3% only with water. A total of 53.6% of students confirmed that they were talked about the importance of handwashing, while 49.4% of students did not practiced handwashing with teachers or in a group with classmates. School staff plays an essential role in practicing hand hygiene. Therefore, for hygiene promotion, schools should undertake activities, such as hygiene education included in the curriculum, extracurricular activities on handwashing, group handwashing facilities, informational materials about handwashing, reminders and posters, regular training of teachers, and teachers’ reminders about washing hands.

### 3.5. Data Collection Tool Assessment

The tool “Surveillance of water, sanitation, and hygiene in schools” was used to collect data. All three methods of data collection within the Tool, namely observation, interview with the school administration, and pupils’ questionnaire, showed high relevance. However, the pupils’ questionnaire was extremely useful for collecting the data from the pupils. The methodology is quite structured, well-organized, and gives insights into the pupils’ access to WASH. In general, the interview with the school administration could be recommended to use as a self-assessment tool. Therefore, it is deemed fit for use. That is why both the school administration and external people could use this tool to assess the workability of the school. When it comes to observation, the teachers responsible for handwashing facilities and handwashing behavior and keeping the infrastructure in proper condition could use it as an internal observation. Out of three data collection tools for assessing access to WASH, the data collected from pupils seem more relevant and could be used considering all questions. By observation, there should be two local positions: one of them responsible for hygiene behavior which could be trained to assess handwashing practices among kids; at the same time, another person could be responsible for maintaining the maintenance and clean conditions and assessing them from time to time. School administration interview is the most relevant for the head of the school to understand the local condition in school. Thus, it can be concluded that this tool could be a good tool for data collection, as various data could be analyzed for different purposes. We believe all three methods of data collection within the tool could be used. However, what should be done most regularly is observing handwashing behavior and cleanliness in the water and sanitation units; less frequently, schools should administer a questionnaire to the pupils, and revision and self-assessment within school administration should take place perhaps once a year.

Concluding, the tool “Surveillance of water, sanitation, and hygiene in schools” could be used at the local and national level to monitor the holistic view of access to WASH and consequently could be used to cover SDG 6 in schools. Furthermore, the data collection methods used in the tool show high relevance for successful use. At the same time, each school could adapt certain parts of the tool.

## 4. Conclusions

The Sustainable Development Goals address the provision of safe water and sanitation for all and increase schools’ ability to offer an effective learning environment, including essential drinking water, sanitation, and hygiene. Despite this, the study reveals that the existence of the required infrastructure is not enough to turn this challenge into an opportunity. This study shows gaps in several vital aspects, such as accessibility, maintenance, operation, education and practices, and student’s satisfaction with the infrastructure. Only through the collaboration between local authorities, school administration, and parents within the community can these basic needs be met. Such cooperation will help to improve the learning environment in schools as well as achieving the SDGs. Consequently, surveying the WASH satisfaction among students and school staff within Kazakh school settings using the tool “Surveillance of water, sanitation, and hygiene in schools” contributes to the effective planning of the WASH system and contributes to the sustainable development of Kazakhstan.

## Data Availability

Data are available in a publicly accessible repository. The data provided in this study will be attached to the article.

## Appendix A

**Table A1:** Pupil’s sample description.

Description	School 1	School 2	School 3	Total
Total pupils	102 (61.5%)	35 (21%)	29 (17.5%)	166 (100%)
Gender:				
Female	50 (49%)	23 (65.7%)	12 (41.4%)	85 (51.2%)
Male	48 (47%)	12 (34.3%)	17 (58.6%)	77 (46.4%)
Missing	4 (3.9%)	-	-	4 (2.4%)
Mean age	10.98 ± 3.3 (SD)	11.6 ± 2.37 (SD)	14.03 ± 2.16 (SD)	11.67 ± 3.14 (SD)
Minimum age	7	7	10	7
Maximum age	17	15	17	17
Classes:				
The 2nd grade	18 (17.6%)	2 (5.7%)	-	20 (12%)
The 3rd grade	21 (20.6%)	3 (8.6%)	-	24 (14.46%)
The 4th grade	15 (14.7%)	4 (11.4%)	-	19 (11.45%)
The 5th grade	4 (3.9%)	5 (14.3%)	3 (10.3%)	12 (7.3%)
The 6th grade	5 (4.9%)	5 (14.3%)	3 (10.3%)	13 (7.8%)
The 7th grade	-	6 (17.1%)	2 (6.9%)	8 (4.8%)
The 8th grade	4 (3.9%)	4 (11.4%)	3 (10.3%)	11 (6.6%)
The 9th grade	7 (6.8%)	5 (14.3%)	7 (24.1%)	19 (11.4%)
The 10th grade	4 (3.9%)	-	5 (17.2%)	9 (5.4%)
The 11th grade	17 (16.7%)	-	5 (17.2%)	22 (13.2%)
Missing	7 (6.8%)	1 (2.9%)	1 (3.4%)	9(5.4%)

**Table A2:** Description of educational organizations.

Description	School 1	School 2	School 3
Type of school management	public	public	public
School program Short (4–6 h) long (7–10 h)	long	long	long
Number of shifts per day	2	2	2
The budget for the operation and maintenance of WASH services	less than 50% of needs of the school	more than 75% of needs of the school	No
Responsibility for the provision, operation, and maintenance of WASH services on the school premises	school administration	local state organizations with school administration	local state organizations with school administration
Total number of students	182	40	58
male	100 (54.9%)	17 (42.5%)	34 (58.6%)
female	82 (45.1%)	23 (57.5%)	24 (42.4%)
Total number of staff	66	30	45
teachers	42	14(46.7%)	17 (37.8%)
administrative staff	6	3 (10%)	3 (8.9%)
operation and maintenance staff	18	13 (43.3%)	25 (55.6%)
The building was built-in	2006	1961	1967
Renovation of WASH systems		2019	2018

**Table A3:** Data analysis triangulation.

Interview	Observation	Pupil Questionnaire
Water: availability and functionality		
The main resource of drinking water	The main resource of drinking water	Availability of drinking water in school
Availability of drinking water during the school year	Currently the availability of drinking water	The location of drinking-water points
Alternative water source	The location of drinking-water points	Availability of water for handwashing
	Number of drinking-water points	
Water: accessibility		
	Accessibility of drinking-water points for young students	Accessibility of drinking-water points for young students
Water: quality of services, operation, and maintenance		
Sufficiency of water for all needs	Quality characteristics of drinking water (color, odor, taste)	Reasons for not drinking water (bad smell, taste, odor)
Quality of water resource	Measures providing water supply at the school level	
Complaints about availability and quality		
Responsibility for drinking-water source		
Sanitation: availability		
Types of toilets/latrines at school	Types of toilets/latrines for pupils	Visiting the toilet in school
Number of currently available toilets	Number of currently available toilets	
Toilet facilities for staff	Toilet facilities for staff	
	Location of the school toilets	
Sanitation: accessibility, privacy, security		
Toilet facility for girls	Accessibility of toilets for young students	Separate toilets for boys and girls
Privacy of the toilet		Not separate toilets for boys and girls as a problem
Issues with the functionality of toilets		Classmate’s bad experience in school toilet
Accessible toilets for all		Personal bad experience in school
The specified time for visiting the toilet		Accessibility of school toilet for smaller pupil
Permanently accessible toilets		
Sanitation: operation and maintenance		
Cleanliness of the school toilet	Cleanliness of the school toilet	Cleanliness of the school toilet
Lighting in the toilet facilities	Lighting in the toilet facilities	Lighting in the toilet facilities
Ventilation in the toilet facilities	Ventilation in the toilet facilities	Opinion about toilet facilities
Heated toilet facility	Heated toilet facility	Toilet paper in the toilet cubicle
Availability of culturally appropriate means for anal cleansing	Availability of culturally appropriate means for anal cleansing	
Waste bins in the toilet facilities	Waste bins in the toilet facilities	
	Cleaning and maintenance schedule records	
Hygiene: availability and functionality, accessibility		
The location of handwashing facilities	The location of handwashing facilities	Washing hands after using the toilet
Availability of soap for handwashing	Availability of soap for handwashing	Availability of soap for handwashing
	Number of handwashing facilities	Availability of handwashing materials
	Availability of warm running water	Accessibility of handwashing facilities for smallest pupil
	Accessibility of handwashing facilities for smallest pupil	
Hygiene: quality of services, operation, and maintenance		
Cleaning and maintenance schedule records	Cleaning and maintenance schedule records	Opinion about handwashing facilities
	Cleanliness of handwashing facilities	
	Hand drying materials	
Water: education and practices		
Allowed time to drink water	Educational materials	Prevalence of drinking water during studying
Location of drinking-water points		Permission to drink water during the classes
		Time for access to drinking water
		Talking about the importance of the drinking water
Sanitation: education and practices		
Reported episodes of bullying or violence in the school toilet facilities	Posters promoting healthy and/or hygienic use of the toilets	Time for visiting the school toilet
Complaints procedure encouraging pupils to report issues in the school toilet facilities		Talking about toilet hygiene and proper hygiene behaviors
Hygiene: education and practices		
Activities for hygiene promotion at the school	Information about handwashing/hand hygiene	The usual time for washing your hands
		The habit of washing your hands, if both water and soap are available
		Talking about the importance of handwashing
		The practice of handwashing with teachers and/or in a group with other pupils
